# Results from the UNITED study: a multicenter study validating the prognostic effect of the tumor–stroma ratio in colon cancer

**DOI:** 10.1016/j.esmoop.2024.102988

**Published:** 2024-04-12

**Authors:** M. Polack, M.A. Smit, G.W. van Pelt, A.G.H. Roodvoets, E. Meershoek-Klein Kranenbarg, H. Putter, H. Gelderblom, A.S.L.P. Crobach, V. Terpstra, G. Petrushevska, G. Gašljević, S. Kjær-Frifeldt, E.M.V. de Cuba, N.W.J. Bulkmans, G.R. Vink, R. Al Dieri, R.A.E.M. Tollenaar, J.H.J.M. van Krieken, W.E. Mesker, UNITED Collaboration, Gordana Petrushevska, Magdalena Bogdanovska, Panche Zdravkoski, Svetozar Antovic, Darko Dzambaz, Panche Karagjozov, Erienne M.V. de Cuba, Frédérique Beverdam, Jan Jansen, Maarten Vermaas, Gorana Gašljević, Sanne Kjær-Frifeldt, Jan Lindebjerg, Maud Strous, Jeroen F. Vogelaar, Nicole W.J. Bulkmans, Joop van Baarlen, Leonie Mekenkamp, Ronald Hoekstra, Mark Sie, Miriam Cuatrecasas, Sara Simonetti, María Teresa Rodrigo, Iván Archilla Sanz, Jose Guerrero Pineda, Natalja E. Leeuwis-Fedorovich, Koen A. Talsma, Ricella M. Souza da Silva, Miangela M. Lacle, Miriam Koopman, Jan Willem T. Dekker, Arjan van Tilburg, Paolo Nuciforo, Xenia Villalobos Alberú, Stefania Landolfi, Adriana Zucchiatti, Emma Witteveen, Arad Bordbar, Mathijs P. Hendriks, René Arensman, Shonali Natu, Noori Maka, Wilma E. Mesker, Rob A.E.M. Tollenaar, Meaghan Polack, Marloes A. Smit, Gabi W. van Pelt, Hein Putter, Elma Meershoek-Kleinenbarg, Annet G.H. Roodvoets, Augustinus S.L.P. Crobach, Hans Gelderblom, Mário Fontes e Sousa, Paula Borralho Nunes, João Cruz, Ana Raimundo, Nelson Silva, Maria J. Brito, Valeska Terpstra, L.M. Zakhartseva, Raed Al Dieri, Jean-François Fléjou, Roger Feakins, Els Dequeker, Geraldine R. Vink, J.Han J.M. van Krieken

**Affiliations:** 1 Department of Surgery, Leiden University Medical Center, Leiden; 2 Clinical Research Center, Department of Surgery, Leiden University Medical Center, Leiden; 3 Department of Biomedical Data Sciences, Leiden; 4 Department of Medical Oncology, Leiden; 5 Department of Pathology, Leiden University Medical Center, Leiden; 6 Department of Pathology, Haaglanden Medical Center, The Hague, The Netherlands; 7 Department of Pathology, Medical Faculty of Ss. Cyril and Methodius University, Skopje, Republic of North Macedonia; 8 Department of Pathology, Onkološki inštitut—Institute of Oncology, Ljubljana, Slovenia; 9 Department of Pathology, Vejle Sygehus–Sygehus Lillebælt, Vejle, Denmark; 10 PATHAN Laboratories, Rotterdam, The Netherlands; 11 Department of Pathology, Spaarne Gasthuis, Haarlem; 12 Department of Medical Oncology, University Medical Center Utrecht, Utrecht University, Utrecht; 13 Department of Research and Development, Netherlands Comprehensive Cancer Organisation (IKNL), Utrecht, The Netherlands; 14 European Society of Pathology, Brussels, Belgium; 15 Department of Pathology, Radboud University Medical Center, Nijmegen, The Netherlands

**Keywords:** colon cancer, tumor microenvironment, tumor–stroma ratio, disease-free survival, pathology

## Abstract

**Background:**

The TNM (tumor–node–metastasis) Evaluation Committee of Union for International Cancer Control (UICC) and College of American Pathologists (CAP) recommended to prospectively validate the cost-effective and robust tumor–stroma ratio (TSR) as an independent prognostic parameter, since high intratumor stromal percentages have previously predicted poor patient-related outcomes.

**Patients and methods:**

The ‘Uniform Noting for International application of Tumor-stroma ratio as Easy Diagnostic tool' (UNITED) study enrolled patients in 27 participating centers in 12 countries worldwide. The TSR, categorized as stroma-high (>50%) or stroma-low (≤50%), was scored through standardized microscopic assessment by certified pathologists, and effect on disease-free survival (DFS) was evaluated with 3-year median follow-up. Secondary endpoints were benefit assessment of adjuvant chemotherapy (ACT) and overall survival (OS).

**Results:**

A total of 1537 patients were included, with 1388 eligible stage II/III patients curatively operated between 2015 and 2021. DFS was significantly shorter in stroma-high (*n* = 428) than in stroma-low patients (*n* = 960) (3-year rates 70% versus 83%; *P <* 0.001). In multivariate analysis, TSR remained an independent prognosticator for DFS (*P* < 0.001, hazard ratio 1.49, 95% confidence interval 1.17-1.90). As secondary outcome, DFS was also worse in stage II and III stroma-high patients despite adjuvant treatment (3-year rates stage II 73% versus 92% and stage III 66% versus 80%; *P* = 0.008 and *P* = 0.011, respectively). In stage II patients not receiving ACT (*n =* 322), the TSR outperformed the American Society of Clinical Oncology (ASCO) criteria in identifying patients at risk of events (event rate 21% versus 9%), with a higher discriminatory 3-year DFS rate (stroma-high 80% versus ASCO high risk 91%). A trend toward worse 5-year OS in stroma-high was noticeable (74% versus 83% stroma-low; *P* = 0.102).

**Conclusion:**

The multicenter UNITED study unequivocally validates the TSR as an independent prognosticator, confirming worse outcomes in stroma-high patients. The TSR improved current selection criteria for patients at risk of events, and stroma-high patients potentially experienced chemotherapy resistance. TSR implementation in pathology diagnostics and international guidelines is highly recommended as aid in personalized treatment.

## Highlights

• The TSR is a cost-effective and robust histological parameter scored on routine hematoxylin–eosin-stained slides.
• This study prospectively validates the independent prognostic effect of the TSR for patients with stage II-III colon cancer.
• Stroma-high tumors (i.e. intratumoral stromal percentage >50%) significantly lead to worse DFS.
• Stroma-high tumors also exhibit chemotherapy resistance, emphasizing a clinical need for new therapy strategies.
• Implementation of the TSR in international guidelines for improved guidance in oncological therapy selection is envisioned.

## Graphical abstract

**Figure:**
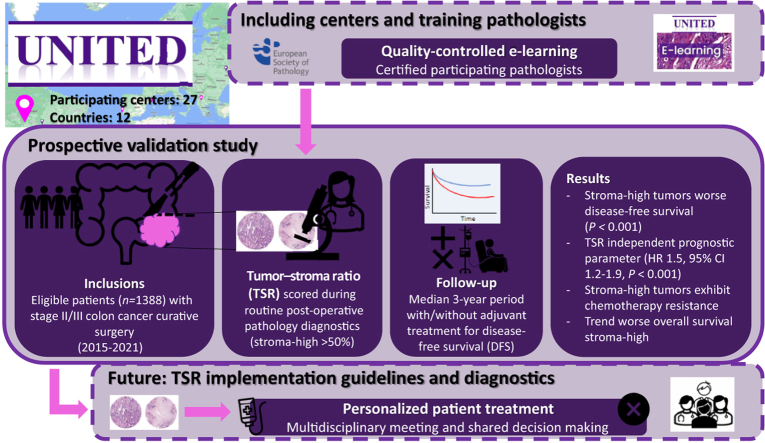

## Introduction

Current treatment guidelines for colon cancer are traditionally based on extent of disease, expressed through the TNM (tumor–node–metastasis) classification, as well as risk assessments for patient outcome and expected benefits of adjuvant chemotherapy (ACT).^1^, ^2^, ^3^, ^4^, ^5^, ^6^ However, the prognostic capacity of TNM staging remains suboptimal. Overtreatment, when patients do not or barely benefit from their ACT, as well as undertreatment, when patients actually could have benefited from additional treatment to prevent recurrences, therefore still occur at high rates.^2^^,^^3^ This supports the clinical need to improve individualized ACT indications upfront through additional prognostic biomarkers. Many pathological parameters have been discovered and implemented in guidelines, like tumor differentiation grade, tumor budding, or microsatellite instability (MSI) status.^2^^,^^3^^,^^7^^,^^8^ However, these mainly focus on the tumor epithelial compartment.

In the past decades, the tumor stroma, a major component within the tumor microenvironment, emerged as an important influencer herein.^9^, ^10^, ^11^, ^12^, ^13^, ^14^ Abundance of intratumoral stroma has been demonstrated to lead to worse patient-related outcomes.^15^^,^^16^ The tumor–stroma ratio (TSR) is a histopathological parameter based on the amount of stroma expressed in percentages compared to the tumor epithelial component, and was initially developed in colon cancer, but has repeatedly been shown to be of prognostic value for almost all epithelial cancers. Patients with stroma-high tumors, i.e. >50% stroma, have a worse disease-free and overall survival (DFS and OS, respectively) than patients with stroma-low tumors, i.e. ≤50% stroma.^17^, ^18^, ^19^, ^20^, ^21^

Implementation of the TSR in international guidelines and pathology diagnostics was advocated to the TNM Evaluation Committee of the Union for International Cancer Control (UICC), and College of American Pathologists (CAP). Although these instances acknowledged the high potential of the TSR as a prognostic parameter, validation was advised, including consensus on scoring of the TSR. Therefore, the present ‘Uniform Noting for International application of Tumor-stroma ratio as Easy Diagnostic tool’ (UNITED) [Dutch Trial Register NL7072; https://clinicaltrialregister.nl/en/trial/23560; International Registered Report Identifier (IRRID): DERR1-10.2196/13464] prospective multicenter study was initiated.^22^

Herein, we hypothesized that patients with stroma-high tumors will have worse outcomes compared with patients with stroma-low tumors. Our primary endpoint was to determine the influence of the TSR on DFS, and secondary endpoints were influence of TSR on benefit of ACT, and on OS. The added value of the TSR in clinical treatment decision making could be, based on this prognostic information, to select patients with stage II stroma-high tumors for ACT, whereas the older patient with comorbidity and a stage III stroma-low tumor could potentially be spared a burdensome and costly treatment. Validation of the TSR will result in unequivocally high-level evidence to accomplish implementation in international guidelines, aiding in shared decision making through improved personalized treatment. Through this UNITED study, we aimed to validate the prognostic effect of the TSR in colon cancer patients.

## Patients and methods

### Patients

The UNITED study was an investigator-driven, prospective, observational, multicenter cohort study, enrolling patients in 27 centers from 12 countries. Approved and contracted centers could only start including after participating pathologists were certified through the official UNITED study e-learning.^23^ Coordination, including contract and database management, quality control, and overall support, was done by the Clinical Research Center from the Leiden University Medical Center (LUMC).

Patients ≥18 years of age with pathological stage II/III colon cancer and who had undergone a complete curative resection (R0) of their primary tumor were eligible. Patients were excluded in case of receiving neoadjuvant treatment, rectal cancer, multiple synchronous tumors, previous malignancies ≤10 years before the current cancer (except basal cell cancer or cervical cancer *in situ*), or any colon cancer in their medical history. Post-operative exclusion criteria were pathological stage I or IV and mortality within 3 months (Supplementary Table S1, available at https://doi.org/10.1016/j.esmoop.2024.102988).

A sample size calculation was carried out previously.^15^^,^^16^ For a 3-year median follow-up period, 114 and 97 recurrences were necessary for sufficient power for stage II and III colon cancer, respectively, requiring 722 and 450 assessable patients in both groups. To obtain this minimum of 1172 inclusions, ∼1500 patients (+25%) were to be registered in total, as inclusions in prospective cohort studies could ultimately be ineligible.^22^

### Materials and tumor–stroma analysis

Diagnostic hematoxylin–eosin (H&E)-stained slides of included patients, on which the T stage of the tumor was already determined, were also used for scoring the TSR through conventional microscopy. All participating pathologists were trained through the official UNITED study e-learning.^23^^,^^24^ This quality-controlled e-learning was supported by the European Society of Pathology (ESP) with official consensus on TSR scoring. High Cohen’s interobserver agreement κ values of >0.70 (at least substantial agreement) were previously observed, proving the reliability and efficiency in teaching the TSR scoring method, also long term.^23^ The stromal percentage was scored on these H&E-stained slides according to the established method of van Pelt et al.,^24^ per 10% increments. Subsequently, categorization using the predefined cut-off value of 50% resulted in stroma-low (≤50%) and stroma-high (>50%) groups, similar to multiple previous studies^15^^,^^20^^,^^24^ (Supplementary Figure S1, available at https://doi.org/10.1016/j.esmoop.2024.102988).

### Statistical analysis

DFS was defined as the time between the date of surgery and the date of first event, i.e. recurrence (locoregional recurrence or distant metastasis) or death (by any cause). In the case of no event, DFS was calculated from the date of surgery until censoring. Although accurate interpretation is only possible after 5 years, we also analyzed the preliminary effect of TSR on OS. OS was defined as the time from the date of surgery until the date of death (by any cause) or until censoring. Censoring took place when patients were disease-free and/or alive at their last follow-up appointment.

The influence of TSR on benefit of ACT was assessed through comparison of TSR categories on DFS with treatment. As ACT is not routinely recommended in stage II colon cancer, the American Society of Clinical Oncology (ASCO) criteria can be used to select those at high risk for events like recurrences, and thus could potentially benefit from this treatment: the stage II-high risk. A pT4 tumor is deemed the most important ASCO risk criterion and indeed generally adhered to in the Netherlands, but also sampling of <12 lymph nodes or emergency operation setting, presence of pathological risk factors like lymphovascular or perineural invasion, and poor tumor differentiation are risk factors.^3^ Firstly, for both pathological stages separately to minimize bias, the influence of TSR will be determined in those receiving ACT to assess potential benefit. Subsequently, recurrence rates will be assessed for the TSR compared to ASCO criteria. To facilitate comparison and grouping of patients, all (sub)stages were recoded to the TNM 5 classification.^25^

Statistical analysis was carried out using the chi-square test between ordinal and nominal variables. Through reversed Kaplan–Meier analysis, median follow-up time was calculated. Survival analyses were carried out using Kaplan–Meier analysis with log-rank tests, and associated number needed to treat tables were added. Hazard ratios (HRs) with associated 95% confidence intervals (CIs) were calculated with the Cox proportional hazards model, using significant variables (*P* < 0.05) from the univariate analysis for the multivariate analysis.

All continuous variables are expressed in medians with interquartile ranges (IQRs), whereas nominal and ordinal variables are stated as number of frequencies and corresponding percentages. Two-tailed *P* values <0.05 were considered statistically significant. Statistical analysis was carried out in collaboration with the Department of Biomedical Data Sciences of the LUMC using IBM SPSS Statistics for Windows, version 29.0 (IBM Corp., Armonk, NY) and figures with GraphPad Prism version 9.3.1 for Windows (GraphPad Software, Boston, MA).

### Data storage

The Clinical Research Center coordinated data storage of the UNITED study, through the worldwide used and highly secured cloud-based platform Castor Electronic Data Capture (Castor EDC; Castor, Amsterdam, The Netherlands).^26^ Collection and supply of electronic case report forms, central monitoring, quality control, and generation of queries within the Castor database were carried out by the Clinical Research Center, leading to high-quality and reusable data. As per protocol, all data and documents are stored for a minimum of 15 years.

### Ethical considerations

The UNITED study protocol was approved by the Medical Research Ethics Committee (MREC) of LUMC. All participating centers had their local MREC approve the protocol before inclusion could commence. In the Netherlands, centers were contracted through the Prospective Dutch ColoRectal Cancer cohort (PLCRC).^27^^,^^28^ Patients from this prospective registration study, fulfilling the UNITED study eligibility criteria and treated in one of the participating centers from 2015 onward, were included. The workflow for retrieval of data through PLCRC is illustrated in Supplementary Figure S2, available at https://doi.org/10.1016/j.esmoop.2024.102988. PLCRC explicitly included the possibility of a cohort-within-a-cohort format in their study design; patients signed broad official informed consent forms for the use of their histopathological data by other studies.^28^ The UNITED study was conducted according to the Declaration of Helsinki (2013).

## Results

Between 8 January 2019 and 9 September 2022, a total of 1537 patients were registered. An overview of inclusion numbers per participating center is provided in Supplementary Table S2, available at https://doi.org/10.1016/j.esmoop.2024.102988. Baseline characteristics of all study patients are added in Supplementary Table S3, available at https://doi.org/10.1016/j.esmoop.2024.102988. Due to the in part prospective nature of the study, 31 patients (2%) were ineligible at registration, e.g. based on medical history with another malignancy ≤10 years of their current colon cancer (*n* = 26). Exclusion followed subsequently in 83 (5%) patients, due to presence of multiple tumors (*n* = 21) or other pathology exclusions like pathological stage 0-I (*n* = 42) or IV colon cancer (*n* = 9). Lastly, 35 (2%) were excluded during follow-up, mostly caused by post-operative mortality within 3 months (*n* = 26).

**Figure 1:**
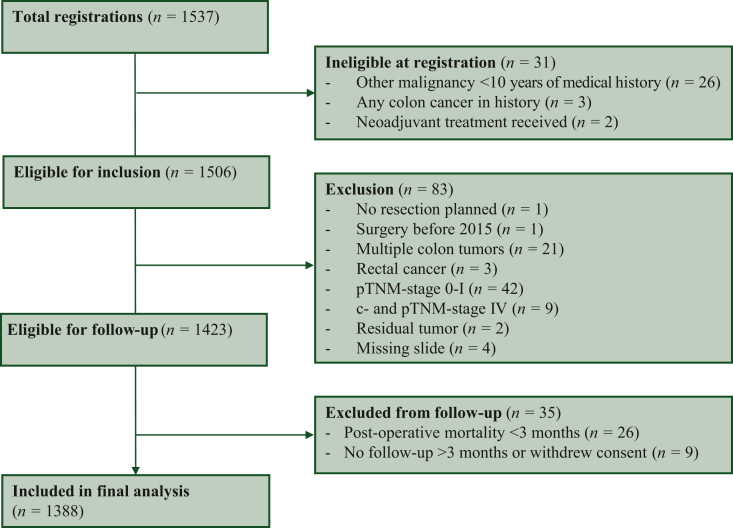
**CONSORT diagram of the UNITED cohort.** CONSORT, Consolidated Standards of Reporting Trials; UNITED, Uniform Noting for International application of Tumor-stroma ratio as Easy Diagnostic tool.

In total, 1388 stage II/III colon cancer patients were included in the final analysis (Figure 1). Of these, 770 patients (55%) were of male sex, and 453 patients were aged ≥75 years (33%). In 1210 (87%) cases, a preoperative endoscopic biopsy was taken, highly indicative of an absence of emergency setting and indicating an elective operation. A total of 723 patients (52%) had stage II colon cancer. The tumor was categorized as stroma-high, i.e. >50% stroma, in 428 patients (31%), which conforms to the previous literature. Patient characteristics of the eligible cohort are summarized in Table 1.

**Table 1:** Baseline characteristics of the eligible patients in the UNITED cohort

Baseline characteristics	UNITED cohort (*N* = 1388)
Sex	
Female	618 (45)
Male	770 (55)
Age at surgery	
Median age (years)	69 (61-77)
≥75 years	453 (33)
Biopsy taken	
Yes, preoperative endoscopy	1210 (87)
Yes, other method^a^	13 (1)
No^b^	165 (12)
Surgery	
Surgery year	2019 (2018-2020)
Pathological stage	
Stage II	723 (52)
Stage III	665 (48)
Lymph nodes	
Examined (in the total group)	20 (15-28)
Positive (in pTNM stage III)^c^	2 (1-4)
Tumor–stroma ratio	
Stroma-low (≤50%)	960 (69)
Stroma-high (>50%)	428 (31)

All variables are given as absolute numbers with associated percentages or medians with interquartile ranges. Sum of percentages can be less or more than 100 due to rounding.

TNM, tumor–node–metastasis.

a Other methods for biopsy are, e.g. during surgery.

b Reasons why biopsy was not taken are, e.g. in emergency setting (obstructive ileus).

c Using the Union for International Cancer Control (UICC) TNM version 8, a tumor deposit (leading to stage N1c) will also lead to a pTNM stage III, also when there are no positive lymph nodes.

Demographics, surgery type, tumor morphology, and differentiation grade were equally distributed amongst stroma-low (*n* = 960) and stroma-high (*n* = 428) groups. Stroma-low tumors were more often right-sided (*P* = 0.049) and had more often <12 lymph nodes sampled (*P* < 0.001); however, in MSI or mismatch repair (MMR) analysis (MSI/MMR), stroma-low tumors were also more prone to MSI or MMR deficiency (MSI/dMMR; *P =* 0.012). Stroma-high tumors ultimately had more risk factors, as these were more often stage III (*P* < 0.001), pT4 stage (*P* < 0.001), and higher pN stage (*P* < 0.001), and more often pathology risk factors like extramural venous invasion were present (*P* < 0.001), illustrating their aggressiveness (Table 2). In merely a small subset (*n =* 153, 11%), mutational status like *KRAS* or *BRAF* was determined, which was not significantly associated to TSR (*P =* 0.150; Supplementary Table S4, available at https://doi.org/10.1016/j.esmoop.2024.102988).

**Table 2:** Analysis of the variables of surgery, pathology, and adjuvant chemotherapy in the eligible UNITED cohort, stroma-low compared with the stroma-high patients

Variables (unit)	Stroma-low (*n* = 960)	Stroma-high (*n* = 428)	*P* value
Sex			0.385^g^
Female	420 (44)	198 (46)	
Male	540 (56)	230 (54)	
Age at surgery—older category			0.185^g^
<75 years	636 (66)	299 (70)	
≥75 years	324 (34)	129 (30)	
Biopsy taken			0.530^g^
Yes	843 (88)	380 (89)	
No^a^	117 (12)	47 (11)	
Surgery type			0.309^h^
Hemicolectomy right	435 (45)	176 (41)	
Hemicolectomy left	121 (13)	59 (14)	
Sigmoidectomy	277 (29)	136 (32)	
Other^b^	127 (13)	57 (13)	
Tumor-sidedness^c^			**0.049**^g^
Right-sided tumor	473 (49)	186 (44)	
Left-sided tumor	487 (51)	241 (56)	
Missing	0 (0)	1 (0)	
Lymph nodes			**0.004**^g^
<12 examined	117 (12)	30 (7)	
≥12 examined	843 (88)	398 (93)	
Pathological tumor (pT) stage^d^			**<0.001**^g^
pT1-3	810 (84)	305 (71)	
pT1	10 (1)	1 (0)	
pT2	61 (6)	6 (1)	
pT3	739 (77)	298 (70)	
pT4	150 (16)	123 (29)	
Pathological nodal (pN) stage^d^			**<0.001**^g^
pN0	541 (56)	182 (43)	
pN1	292 (30)	152 (36)	
pN2	127 (13)	94 (22)	
Tumor morphology			0.303^g^
Adenocarcinoma	855 (89)	390 (91)	
Mucinous carcinoma	90 (9)	33 (8)	
Other, including signet cell carcinoma	15 (2)	5 (1)	
Differentiation grade^e^			0.062^g^
Well–moderate	809 (84)	378 (88)	
Poor–undifferentiated	125 (13)	41 (10)	
Grade cannot be assessed	26 (3)	9 (2)	
Pathology risk factors^f^			**<0.001**^g^
No pathology risk factors present	566 (59)	197 (46)	
Presence of ≥1 pathology risk factor	394 (41)	231 (54)	
Extramural venous invasion (EMVI)			
Not reported	162 (17)	55 (13)	
Reported, of which	798 (83)	373 (87)	**<0.001**^g^
Yes (EMVI+)	86 (9)	98 (23)	
No (EMVI−)	712 (74)	275 (64)	
Venous invasion			
Not reported	99 (10)	56 (13)	
Reported, of which	861 (90)	372 (87)	**<0.001**^g^
Yes (V1)	100 (12)	69 (19)	
No (V0)	761 (88)	303 (81)	
Lymphatic invasion			
Not reported	28 (3)	20 (5)	
Reported, of which	932 (97)	408 (95)	0.193^g^
Yes (L1)	304 (33)	148 (36)	
No (L0)	628 (67)	260 (64)	
Perineural invasion			
Not reported	423 (44)	186 (44)	
Reported, of which	537 (56)	242 (57)	**<0.001**^g^
Yes (Pn1)	68 (13)	58 (24)	
No (Pn0)	469 (87)	184 (43)	
Microsatellite instability/mismatch repair (MMR) status			
Not determined	439 (46)	213 (50)	
Determined, of which	521 (54)	215 (50)	**0.012**^g^
Microsatellite stable (MSS)/MMR proficient (pMMR)	403 (77)	184 (86)	
Microsatellite instable (MSI)/MMR deficient (dMMR)	118 (23)	31 (14)	
Pathological TNM stage			**<0.001**^g^
Stage II	541 (56)	182 (43)	
Stage III	419 (44)	246 (57)	
Adjuvant chemotherapy—received			**<0.001**^g^
No	539 (56)	174 (41)	
Yes	421 (44)	254 (59)	
Adjuvant chemotherapy—per pathological TNM stage			**<0.001**^g^
Stage II + no adjuvant therapy	434 (45)	125 (29)	
Stage II + adjuvant therapy	107 (11)	57 (13)	
Stage III + adjuvant therapy	314 (33)	197 (46)	
Stage III + no adjuvant therapy	105 (11)	49 (11)	

All variables are given as absolute numbers with associated percentages or medians with interquartile ranges. Sum of percentages can be less or more than 100 due to rounding. Bold indicates significance, when *P* < 0.05.

N/A, not applicable; TNM, tumor–node–metastasis.

a Reasons why biopsy was not taken are, e.g. in emergency setting (obstructive ileus).

b Other operation types include a (sub)total colectomy, high anterior resection, or transversectomy.

c A right-sided tumor is defined as a colon carcinoma in the cecum, colon ascendens, flexura hepatica, or colon transversum.

d Different versions of the Union for International Cancer Control (UICC) TNM classification were used. Here, all variables are converted to the UICC TNM version 5 (1997).

e Differentiation grade is variously registered as separate or combined subgroups; this is then categorized into combined grades, i.e. well–moderate or poor–undifferentiated.

f Pathology risk factors are stated in Table 2. Presence of a risk factor is defined as at least one of registered risk factors. Absence is the absence of registered risk factors, as not all risk factors are registered.

g Calculated using the chi-square test.

h Calculated using the chi-square test for the three most common and here presented operation types: hemicolectomy right, hemicolectomy left, and sigmoidectomy.

**Figure 2:**
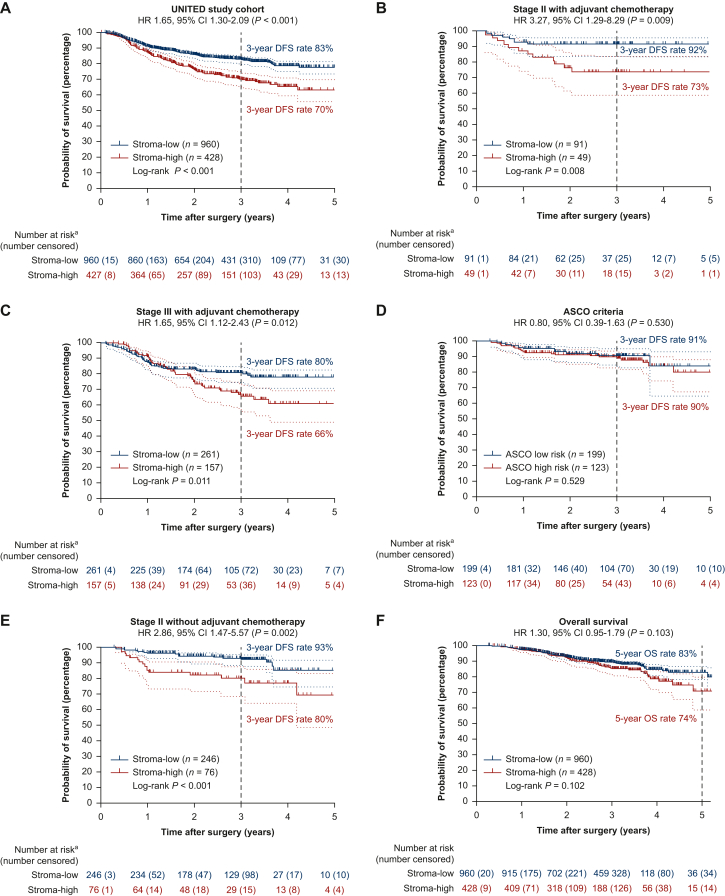
**Disease-free survival effect of TSR in the UNITED cohort and subgroup analyses.** (A) Kaplan–Meier analysis and log-rank test showing worse 3-year disease-free survival rates for stroma-high patients in the whole UNITED cohort (70% versus 83%, respectively; *P* < 0.001). (B) Kaplan–Meier analysis with log-rank test in stage II patients receiving adjuvant chemotherapy, illustrating the worse 3-year survival rates for stroma-high patients despite treatment, indicating potential resistance to adjuvant chemotherapy (stroma-high 73% versus stroma-low 92%; *P* = 0.008). (C) Kaplan–Meier analysis with log-rank test in stage III patients receiving adjuvant chemotherapy, again illustrating the worse 3-year survival rates for stroma-high patients despite treatment (stroma-high 66% versus stroma-low 80%; *P* = 0.011). (D) The ASCO criteria (high risk versus low risk) not distinguishing any disease-free survival difference (high risk 91% versus low risk 90%; *P* = 0.529). (E) Kaplan–Meier analysis with log-rank test in stage II patients not receiving adjuvant chemotherapy, showing significant worse 3-year survival rates in stroma-high patients compared to stroma-low patients (stroma-high 80% versus stroma-low 93%; *P* < 0.001). (F) Kaplan–Meier analysis with log-rank test, showing overall worse survival in the stroma-high groups despite the short median follow-up of 3 years instead of 5 years, with the curves already diverging at 3 years. The 5-year overall survival rates are 74% versus 83%, respectively (*P* = 0.102). ASCO, American Society for Clinical Oncology; TNM, tumor–node–metastasis; TSR, tumor–stroma ratio; UNITED, Uniform Noting for International application of the Tumor-stroma ratio as Easy Diagnostic tool.^a^For disease-free survival, the number of patients starting can be lower due to missing data.

Median follow-up time was 36.2 months (95% CI 35.9-36.5 months) at the time of database lock (31 January 2023), and comparable between both groups (*P* = 0.469) (Supplementary Figure S3, available at https://doi.org/10.1016/j.esmoop.2024.102988). Generally, follow-up was according to daily clinical practice, differing per country and center, but approximately at 6, 12, and 24-36 months post-operatively. A total of 286 events occurred, of which 123 in the stroma-high group (29% of stroma-high patients; *P* < 0.001). Mostly, this concerned distant metastases (92 stroma-high patients, 75% of events) (Supplementary Table S4, available at https://doi.org/10.1016/j.esmoop.2024.102988). Hence, a statistically significantly worse DFS was observed in stroma-high patients with 3-year DFS rates of 70%, compared to 83% in stroma-low patients (HR 1.78, 95% CI 1.41-2.26; *P* < 0.001; Figure 2). In multivariate analysis, after correcting for significant univariate variables, DFS remained worse for stroma-high compared to stroma-low patients, confirming the independent prognostic effect of the TSR on DFS (HR 1.49, 95% CI 1.17-1.90; *P* < 0.001) (Table 3). Forest plots for these univariate and multivariate analyses are provided in Supplementary Figures S4 and S5, available at https://doi.org/10.1016/j.esmoop.2024.102988.

**Table 3:** Univariate and multivariate analysis of disease-free survival in the UNITED cohort with Cox regression analysis

Variable (unit)	Univariate analysis		*P* value	Multivariate analysis		*P* value
	Disease-free survival			Disease-free survival		
	Hazard ratio	95% confidence interval		Hazard ratio	95% confidence interval	
Sex						
Female	1	—	0.753	—	—	—
Male	1.04	0.82-1.31	—	—	—	—
Age at surgery—older category						
<75 years	1	—	**0.036**	1	—	**<0.001**
≥75 years	1.29	1.02-1.63	—	1.54	1.21-1.96	—
Biopsy taken						
Yes	1	—	**<0.001**	1	—	**<0.001**
No^a^	2.60	1.96-3.46	—	2.33	1.72-3.16	—
Tumor-sidedness^b^						
Left-sided tumor	1	—	0.404	—	—	—
Right-sided tumor	0.91	0.72-1.14	—	—	—	—
Lymph nodes						
≥12 examined	1	—	**0.009**	1	—	0.067
<12 examined	1.54	1.11-2.14	—	1.38	0.98-1.95	—
pT stage^c^						
pT1-3	1	—	**<0.001**	1	—	**<0.001**
pT4	2.25	1.76-2.89	—	1.59	1.22-2.07	—
pN stage^c^						
pN0	1	—	**<0.001**	1	—	**<0.001**
pN1	1.88	1.43-2.48	—	1.71	1.28-2.30	—
pN2	3.33	2.48-4.47	—	2.66	1.91-3.70	—
Tumor morphology						
Adenocarcinoma	1	—	0.514	—	—	—
Other, including mucinous and signet cell carcinoma	1.13	0.79-1.62	—	—	—	—
Differentiation grade						
Well–moderate	1	—	0.191	—	—	—
Poor–undifferentiated	1.25	0.90-1.75	—	—	—	—
Pathology risk factors						
No pathology risk factors present^d^	1	—	**<0.001**	1	—	**0.018**
Presence of ≥1 pathology risk factor	2.17	1.71-2.76	—	1.38	1.06-1.81	—
Adjuvant chemotherapy—received						
No	1	—	0.267	—	—	—
Yes	1.14	0.90-1.44	—	—	—	—
Tumor–stroma ratio						
Stroma-low	1	—	**<0.001**	1	—	**0.001**
Stroma-high	1.78	1.41-2.26	—	1.49	1.17-1.90	—
Microsatellite stability^e^						
Microsatellite instable (MSI)	1	—	0.070	—	—	—
Microsatellite stable (MSS)	1.55	0.97-2.48	—	—	—	—

All variables are given as absolute numbers with associated percentages or medians with interquartile ranges. Bold indicates significance, when *P* < 0.05.

a Reasons why biopsy was not taken are, e.g. in emergency setting (obstructive ileus).

b A right-sided tumor is defined as a colon carcinoma in the coecum, colon ascendens, flexura hepatica, or colon transversum.

c Different versions of the Union for International Cancer Control (UICC) TNM classification were used. Here, all variables are converted to the UICC TNM version 5 (1997).

d Pathology risk factors are stated in Table 2. Presence of a risk factor is defined as at least one of registered risk factors. Absence is the absence of registered risk factors, as not all risk factors are registered.

e Dependent covariate, excluded in multivariate analysis also due to insignificance and minority of patients determined.

The effect of TSR on DFS per stage is illustrated in Supplementary Figure S6, available at https://doi.org/10.1016/j.esmoop.2024.102988. In stage II, the worse DFS for stroma-high patients remained significant with 3-year DFS rates of 77% versus 91% in stroma-low (*P <* 0.001), but for stage III this was not the case (3-year DFS rates 65% versus 72%, respectively; *P* = 0.055). However, a significant bias occurred in stage III patients due to the difference in median age in stage III for patients who received ACT (65 years, IQR 57-72 years) and those who did not (79 years, IQR 71-84 years; Student’s independent *t*-test *P* < 0.001). As this led to skewed results, stratification for age was deemed necessary here. After stratifying for age (<75 years), stroma-high stage III colon cancer led to significantly worse 3-year DFS rates as well (64% versus 78%; *P* = 0.008). The predictive potential of the TSR on benefit of ACT was investigated as secondary outcome in these groups.

A total of 676 patients (49%) of the UNITED study started with ACT, mostly intravenous oxaliplatin combined with oral capecitabine (CAPOX/XELOX; *n* = 394, 58%). A detailed overview of treatment regimens is given in Supplementary Table S5, available at https://doi.org/10.1016/j.esmoop.2024.102988. Although treatment guidelines can differ between countries, centers, and can be dependent on the decision of the physician and/or patient, we also looked within stages at those receiving ACT or not, to ascertain the benefit of patients who received additional treatment on DFS in reducing risk of recurrences.

Specifically, we analyzed potential added benefit from ACT in stage II and III patients and influence of the TSR herein on DFS, after correction for age (<75 years). Within the stage II patients who did receive ACT (*n* = 140), mostly stage II-high risk, a significantly worse DFS was seen (3-year DFS rates stage II with ACT 73% stroma-high versus 92% stroma-low; *P =* 0.008). In the stage III group receiving standard-of-care ACT (*n* = 418), 3-year DFS rates were significantly worse for stroma-high patients than their stroma-low counterparts despite their ACT, too (66% versus 80%; *P* = 0.011). (Figure 2B and C). This illustrates that stroma-low patients could benefit from ACT, but that stroma-high patients exhibit a lack of benefit or even potential resistance to ACT. Supplementary Figure S7A and B, available at https://doi.org/10.1016/j.esmoop.2024.102988, shows per TSR category the different groups, with indeed worse DFS rates in all stroma-high groups not significantly increasing despite ACT (*P =* 0.080) compared to stroma-low patients (*P <* 0.001).

To assess which parameter could potentially have identified more patients at risk for an event, in-depth analysis on the subgroup of stage II patients <75 years of age not receiving ACT (*n =* 322) was carried out, comparing the TSR to the ASCO criteria. According to the ASCO criteria, in these stage II patients, 123 (38%) patients fulfilled one or more high-risk criteria. In this ASCO high-risk group, a 9% (*n* = 11) event rate was observed, illustrating the percentage of undertreated patients. In the ASCO low-risk group, however, in 24 cases (12%) an event occurred. The ASCO criteria did thus not correctly identify patients at risk for events or show differences in DFS rates (*P* = 0.383 in Supplementary Table S6, available at https://doi.org/10.1016/j.esmoop.2024.102988; 3-year DFS rates 90% low risk versus 91% high risk, *P* = 0.529 in Figure 2D, respectively). For TSR analysis, in this group of stage II patients not receiving ACT (*n* = 322, 246 stroma-low and 76 stroma-high), a total of 35 events (11% event rate) occurred. Of these events, 16 occurred in the stroma-high group (21% event rate in the stroma-high group) and 19 in the stroma-low group (8% event rate in the stroma-low group; *P =* 0.001). DFS rates plotted in this subgroup show similar differences (80% versus 93%; *P* < 0.001) (Figure 2E). Compared to the ASCO criteria, the TSR thus identified an additional 12% patients at risk for events (21% versus 9%) with a 91% 3-year DFS rate for ASCO high-risk patients in comparison to the 80% in stroma-high patients.

Although the UNITED study was powered specifically for DFS with a median 3-year follow-up period, as secondary endpoint the preliminary effect of the TSR on 5-year OS was estimated. A total of 163 deaths were recorded, of which 61 were in the stroma-high group (14% of stroma-high patients; *P* < 0.001). In the plotted Kaplan–Meier analysis and log-rank analysis, effect on OS was not statistically significant, despite the relatively short median follow-up (HR 1.30, 95% CI 0.95-1.79; *P =* 0.103). At 5 years, OS rates of 74% in stroma-high versus 83% in stroma-low colon cancer, respectively, were observed (*P* = 0.102; Figure 2).

## Discussion

The UNITED study was initiated to prospectively validate the TSR as a prognostic parameter in colon cancer patients. This study not only confirms that patients with stroma-high tumors indeed have significantly worse DFS, but also proves that this effect is independent from other prognostic high-risk parameters, such as sampling of <12 lymph nodes and pathological T stage or N stage. Moreover, the TSR also outperformed the current ASCO criteria in identification of stage II colon cancer patients at risk for events. We had hypothesized that fit, stage II stroma-high patients could benefit from additional ACT and frail stage III stroma-low patients with better outcomes could perhaps be spared this treatment. However, our secondary findings contrarily indicate that stroma-low patients benefit from ACT, whereas stroma-high patients do not and thus could actually be considered to not be selected for ACT. This study illustrates the aggressive behavior of stroma-high tumors and the potential resistance to (neo)adjuvant treatment of tumor stroma, which was also noticed in other studies by our research group.^17^^,^^29^, ^30^, ^31^

Despite the relatively short follow-up period of 3 instead of 5 years, a trend toward worse OS is already seen for stroma-high colon cancer. The curves diverge after 3 years, probably since most events, i.e. recurrences, occur within the first 3 years after primary diagnosis.^2^ Additionally, due to the increase in sequential treatment options that may have extended survival in patients with recurrences or metastases, events mainly lead to an effect on DFS but not immediately on OS.^2^^,^^3^^,^^32^ Therefore, we aim to collect longer follow-up data of UNITED study patients in the future, to adequately evaluate the effect of the TSR on OS after 5-10 years.

In 2007, our research group was the first to describe the phenomenon of a high intratumoral stroma percentage and the associated worse patient-related outcomes in colon cancer.^15^ Since then, much research has been carried out regarding the role of tumor stroma, aiming to elucidate the biological mechanism. The intricate and dynamic tumor–stroma crosstalk has been observed to include the cancer-associated fibroblasts as important players, potentially also enabling the seed-and-soil principle of Paget and causing stromal metastases in lymph nodes.^14^^,^^33^ The TSR can be scored on these metastases as well, and patients with stroma-high primary tumors and stroma-high lymph node metastases have been observed to have the worst survival.^34^^,^^35^ Even small lymph nodes ≤5 mm in diameter, during routine radiologic imaging not suspected of malignancy, can contain metastases. Scoring the TSR in lymph nodes in the future, as well as more research on improving positive lymph node detection, is pertinent for an even more tailored treatment.^33^

Many biomarkers have emerged in the past decades, as researchers are aiming to better predict tumor behavior and patient outcomes. One such emerging biomarker is liquid biopsy, measuring circulating tumor DNA strands in blood as a marker for minimal residual disease.^36^^,^^37^ Even more of interest is the study on tumor stromal liquid biopsy panels, capturing the tumor microenvironment.^38^^,^^39^ However, not only do these increasing number of biomarkers add to existing high work load and are time consuming, often, more patient material or resources are necessary. Moreover, some biomarkers have variance in analyses, like the consensus molecular subtypes (CMS). CMS type 4, the mesenchymal type, mostly covers stroma-high tumors, but low reproducibility prohibited accurate analysis.^40^^,^^41^ Also tumor budding, now implemented in guidelines as an additional prognosticator, is known to have a less optimal interobserver agreement.^42^ Although the TSR strictly does not capture the qualitative histopathological heterogeneity of the complete tumor–stroma entity, the UNITED study shows that the quantitatively determined TSR still is an independent prognostic parameter, robust and simple, determined by pathologists during routine diagnostic microscopy assessment under 2 min without extra resources or costs, and therefore cost-effective.^15^^,^^17^^,^^20^^,^^24^ Potentially additional analyses, e.g. organization or maturity determination, can be done and would be of interest to carry out on UNITED study material for further characterization of the tumor stroma.^10^^,^^43^

Also, the tumor immune microenvironment has been proven to affect tumor behavior, as for instance seen in the Immunoscore.^44^ High influx of tumor-infiltrating immune cells (TIICs) is often indeed correlated with decreased rates of recurrence. Hence, increasing amounts of research demonstrate the high potential of certain immunotherapeutic regimens in cancer. Although there are various potential analyses to ascertain the tumor immune microenvironment, often also requiring additional resources and patient material like with the Immunoscore method, future analysis of TIICs in all UNITED study patients would be of interest.^44^ Previous research namely shows that the amount of influx of TIICs combined with TSR forms a potentially even more accurate prognosticator, as stroma-high/immune-low tumors are associated with the worst patient-related outcomes.^45^

There are some limitations to the present study. MSI/MMR status has only been obligatory in current treatment guidelines and thus was not determined in the included older first half of patients.^2^ Additionally, our analyses on ACT, as the UNITED study was not powered or set up for this specifically, may be biased despite stratification for risk factors like age, e.g. due to the powerful a priori prognostic effect. Although the association seen in our analyses is already significant, we are currently initiating a well-balanced, matched cohort specifically powered for this endpoint to confirm the apparent resistance to ACT in stroma-high patients. Also, median 3 years of follow-up is relatively short, but fulfills the standard to adequately ascertain DFS, as previously taken into account in our power calculation, and can even be interpreted as a valid surrogate for OS.^22^^,^^46^

Strengths of this study include foremost the prospective nature, to minimize bias and adequately evaluate the causal effect of the TSR on survival. After the initial proposal of implementation of the TSR in TNM-based guidelines to the UICC and CAP, various supportive collaborations have been established for this study. Participating pathologists were trained with the reliable and quality-controlled UNITED study e-learning.^22^^,^^23^ Support grew, ultimately leading to the completion of this study. Not only has the TSR thus been validated, but the UNITED study also led to the foundation of an international system for pathologists to potentially implement the TSR in daily routine diagnostics. Moreover, international guidelines regarding ACT can be re-evaluated. As stroma-high patients have a worse DFS and exhibit resistance to ACT, they could be discussed in multidisciplinary settings and potentially be spared this treatment.

As part of this study, centers were requested to send scans of the scored H&E-stained slides. This collection will be used for future development of artificial intelligence algorithms for TSR automatization. Not only can the interobserver agreement be increased even more, also analysis of difficult cases with, for instance, high amounts of mucin or necrosis can be facilitated, and automatization supports the increasing interest in digital pathology as well, including possibilities for even further research. Deep learning models especially can discern even more and potentially novel features in the tumor stroma, e.g. stromal organization like previously analyzed by our research group, and correlations to patient-related outcomes can be assessed.^43^,^47^,^48^

Importantly, future studies should focus on tumor stroma-targeted therapeutic regimens or strategies, as this study shows a lack of benefit of stroma-high colon cancer to ACT, revealing a clear clinical need for new treatment options for these patients. Similar to ACT, tumors with high amounts of tumor stroma also have been observed to respond less to immunotherapeutic strategies.^49^ Although confirming this potential resistance to adjuvant treatment in a powered series is necessary, fundamental research and pharmacological phase I studies should be initiated to uncover specific targets.

## Conclusions

In conclusion, the UNITED study hereby unequivocally confirms the independent prognostic effect of the TSR on DFS in colon cancer patients as per request of the UICC and CAP. As stroma-high patients have worse DFS and appear to benefit less from ACT than stroma-low patients, the TSR can herein aid in clinical shared decision making and personalized treatment. Therefore, implementation of the TSR in standard-of-care pathology diagnostics and reporting in addition to currently used elements as the TNM classification and ultimately in international guidelines is highly recommended.
