# Supplementary material for: Results from the UNITED study: a multicenter study validating the prognostic effect of the tumor–stroma ratio in colon cancer
Source: ESMO Open. 2024 Apr 12;9(4):102988. doi: 10.1016/j.esmoop.2024.102988 (PMC11033069; doi:10.1016/j.esmoop.2024.102988)
Supplement: Supplementary Appendix [file mmc1.docx]

**Appendix Table 1. Participating centres and collaborative investigators**

| Location (country) | Institute | Investigator (department) |
| --- | --- | --- |
| Skopje (Macedonia) | Medical Faculty of Ss. Cyril and Methodius University | Gordana Petrushevska# (pathology)  Magdalena Bogdanovska (pathology)  Panche Zdravkoski (pathology)  Svetozar Antovic (surgery)  Darko Dzambaz (surgery)  Panche Karagjozov (surgery) |
| Rotterdam region (Netherlands) | PATHAN Laboratories$ *(Franciscus Gasthuis & Vlietland; Admiraal De Ruyter Ziekenhuis; IJsselland Ziekenhuis)* | Erienne M.V. de Cuba#* (pathology)  Frédérique Beverdam# (surgery; Franciscus Gasthuis & Vlietland)  Jan Jansen# (surgery; Admiraal de Ruyter Ziekenhuis)  Maarten Vermaas# (surgery; IJsselland Ziekenhuis) |
| Ljubljana (Slovenia) | Onkološki Inštitut | Gorana Gašljević# (pathology) |
| Vejle (Denmark) | Vejle Sygehus – Sygehus Lillebælt | Sanne Kjær-Frifeldt# (pathology)  Jan Lindebjerg (pathology) |
| Venlo (Netherlands) | VieCuri Medisch Centrum | Maud Strous# (pathology)  Jeroen F. Vogelaar (surgery) |
| Hoofddorp and Haarlem (Netherlands) | Spaarne Gasthuis$ | Nicole W.J. Bulkmans#* (pathology) |
| Enschede region (Netherlands) | LabPON$ *(Medisch Spectrum Twente; Ziekenhuisgroep Twente; ZorgSaam Terneuzen)* | Joop van Baarlen# (pathology; retired)  Leonie Mekenkamp (medical oncology; Medisch Spectrum Twente)  Ronald Hoekstra (medical oncology; Ziekenhuisgroep Twente)  Mark Sie (medical oncology; ZorgSaam Terneuzen) |
| Barcelona (Spain) | Hospital Clinic | Miriam Cuatrecasas# (pathology)  Sara Simonetti (pathology)  María Teresa Rodrigo (pathology)  Iván Archilla Sanz (pathology)  Jose Guerrero Pineda (pathology) |
| Deventer (Netherlands) | Deventer Ziekenhuis$ | Natalja E. Leeuwis-Fedorovich# (pathology)  Koen A. Talsma (surgery) |
| João Pessoa (Brazil) | Napoleão Laureano Hospital | Ricella M. Souza da Silva# (pathology) |
| Utrecht (Netherlands) | Universitair Medisch Centrum Utrecht$ | Miangela M. Lacle# (pathology)  Miriam Koopman (medical oncology) |
| Delft (Netherlands) | Reinier de Graaf Gasthuis$ | Jan Willem T. Dekker# (surgery)  Arjan van Tilburg (pathology) |
| Barcelona (Spain) | Vall d’Hebron Institute of Oncology | Paolo Nuciforo# (pathology)  Xenia Villalobos Alberú (pathology)  Stefania Landolfi (pathology)  Adriana Zucchiatti (pathology) |
| Alkmaar (Netherlands) | Symbiant Laboratories$ *(Noordwest Ziekenhuisgroep Alkmaar)* | Emma Witteveen# (pathology)  Arad Bordbar (pathology)  Mathijs P. Hendriks (medical oncology) |
| Amersfoort (Netherlands) | Meander Medisch Centrum$ | René Arensman# (pathology) |
| Hardwick (United Kingdom) | University Hospital of North Tees | Shonali Natu# (pathology) |
| Glasgow regio (United Kingdom) | NHS Greater Glasgow and Clyde | Noori Maka# (pathology) |
| Leiden (Netherlands) | Leids Universitair Medisch Centrum | Wilma E. Mesker# (surgery)  Rob A.E.M. Tollenaar (surgery)  Meaghan Polack (surgery)  Marloes A. Smit (surgery)  Gabi W. van Pelt (surgery)  Hein Putter (biomedical data sciences)  Elma Meershoek-Kleinenbarg (clinical research center, surgery)  Annet G.H. Roodvoets (clinical research center, surgery)  Augustinus S.L.P. Crobach (pathology)  Hans Gelderblom (medical oncology) |
| Lisbon (Portugal) | Hospital CUF Tejo | Mário Fontes e Sousa# (medical oncology)  Paula Borralho Nunes (pathology)  João Cruz (pathology)  Ana Raimundo (medical oncology)  Nelson Silva (surgery) |
| Almada (Portugal) | Hospital Garcia de Orta | Maria J. Brito# (pathology) |
| The Hague (Netherlands) | Haaglanden Medisch Centrum | Valeska Terpstra# (pathology) |
| Kiev (Ukraine) | Bogomolets - Kyiv Oncology Center | L.M. Zakhartseva (pathology) |
| Brussels (Belgium) | European Society for Pathology | Raed Al Dieri (pathology)  Jean-François Fléjou (pathology)  Roger Feakins (pathology)  Els Dequeker (pathology) |
| Utrecht (Netherlands) | Netherlands Comprehensive Cancer Organisation (IKNL)$ | Geraldine R. Vink (research and development) |
| Nijmegen (Netherlands) | Radboud University Medical Center | J. Han J.M van Krieken (pathology) |

# Local principal investigator.

$ Part of the PLCRC collaboration.

* Currently employed elsewhere.
