# Supplementary material for: Results from the UNITED study: a multicenter study validating the prognostic effect of the tumor–stroma ratio in colon cancer
Source: ESMO Open. 2024 Apr 12;9(4):102988. doi: 10.1016/j.esmoop.2024.102988 (PMC11033069; doi:10.1016/j.esmoop.2024.102988)
Supplement: Supplementary Material [file mmc2.pdf]

ONLINE SUPPLEMENTARY MATERIAL

Table of contents

Supplementary Table 1. Final inclusion and exclusion criteria of the UNITED study…………….…………. …2

Supplementary Figure 1. Examples of the tumour-stroma ratio.…………………………………….…….….…..3

Supplementary Figure 2. Workflow patient inclusion through PLCRC collaboration…………………….….……4

Supplementary Table 2. Participating centres and collaborative investigators……………………………..….5

Supplementary Table 3. Baseline characteristics of the total UNITED cohort…………………………………….8

Supplementary Figure 3. Median follow-up time…………………………………………………………….....9

Supplementary Table 4. General overview of patient outcomes …………………………………………..….10

Supplementary Figure 4. Forest plot of the effect of risk factors on disease-free survival (univariate analysis)...13

Supplementary Figure 5. Forest plot of the effect of risk factors on disease-free survival (multivariate analysis).14

Supplementary Figure 6. TSR on Disease-free survival per TNM-stage…………………………………………15

Supplementary Table 5. Detailed overview of the adjuvant chemotherapy regimens and durations…………….16

Supplementary Figure 7. DFS per TSR category and benefit of adjuvant treatment per TNM-stage……………18

Supplementary Table 6. Overview of ASCO criteria and TSR categories..………………………………………19

Supplementary Table 7. Data sharing statement…………………………………………………..…………..20

Supplementary Table 1. Final inclusion and exclusion criteria of the UNITED study

UNITED study	Criteria
**Inclusion criteria** *(eligible for inclusion and follow-up)*	• Operation date after 2015<br>• Histologically proven colon cancer<br>• Pathological stage II (T3-4, N0, M0) or III (every T, N1-2, M0)<br>• Tumor-stroma ratio score<br>• Age ≥ 18 years and signed informed consent
**Exclusion criteria** *(ineligible)*	• Neoadjuvant therapy<br>• Other malignancy 10 year prior to current colon cancer (except basal cell carcinoma or cervix carcinoma in situ) or in the complete medical history a colon carcinoma<br>• Rectal cancer<br>• Multiple synchronous malignant colon cancer<br>• No complete curative resection (R1 of R2 resection)<br>• Postoperative mortality within 3 months of operation

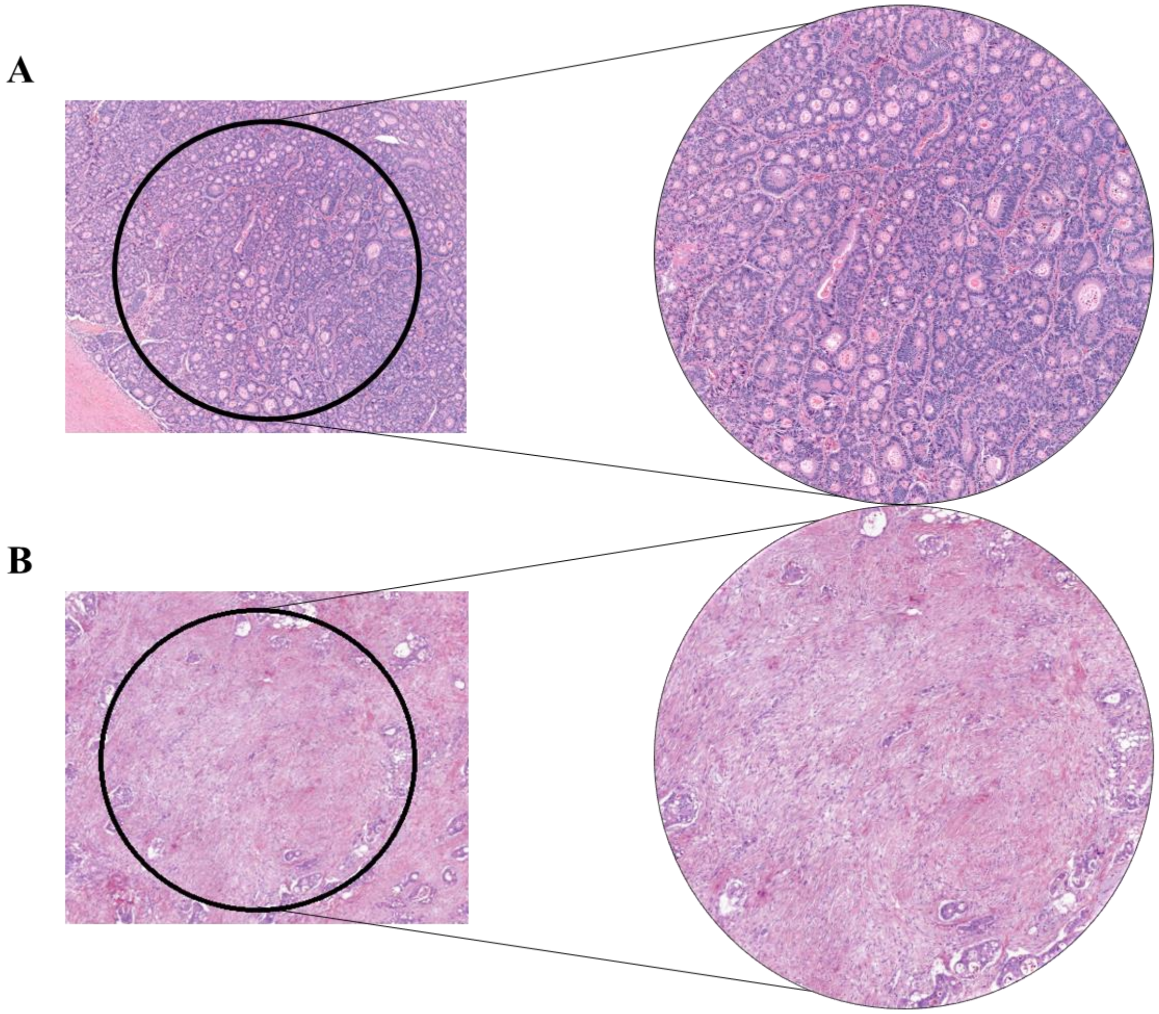

Supplementary Figure S1. Zoomed in examples of the scoring of the tumor-stroma ratio on hematoxylin and eosin-stained slides. (A) Stroma-low (≤50%) colon cancer; (B) Stroma-high (>50%) colon cancer. 10x magnification.

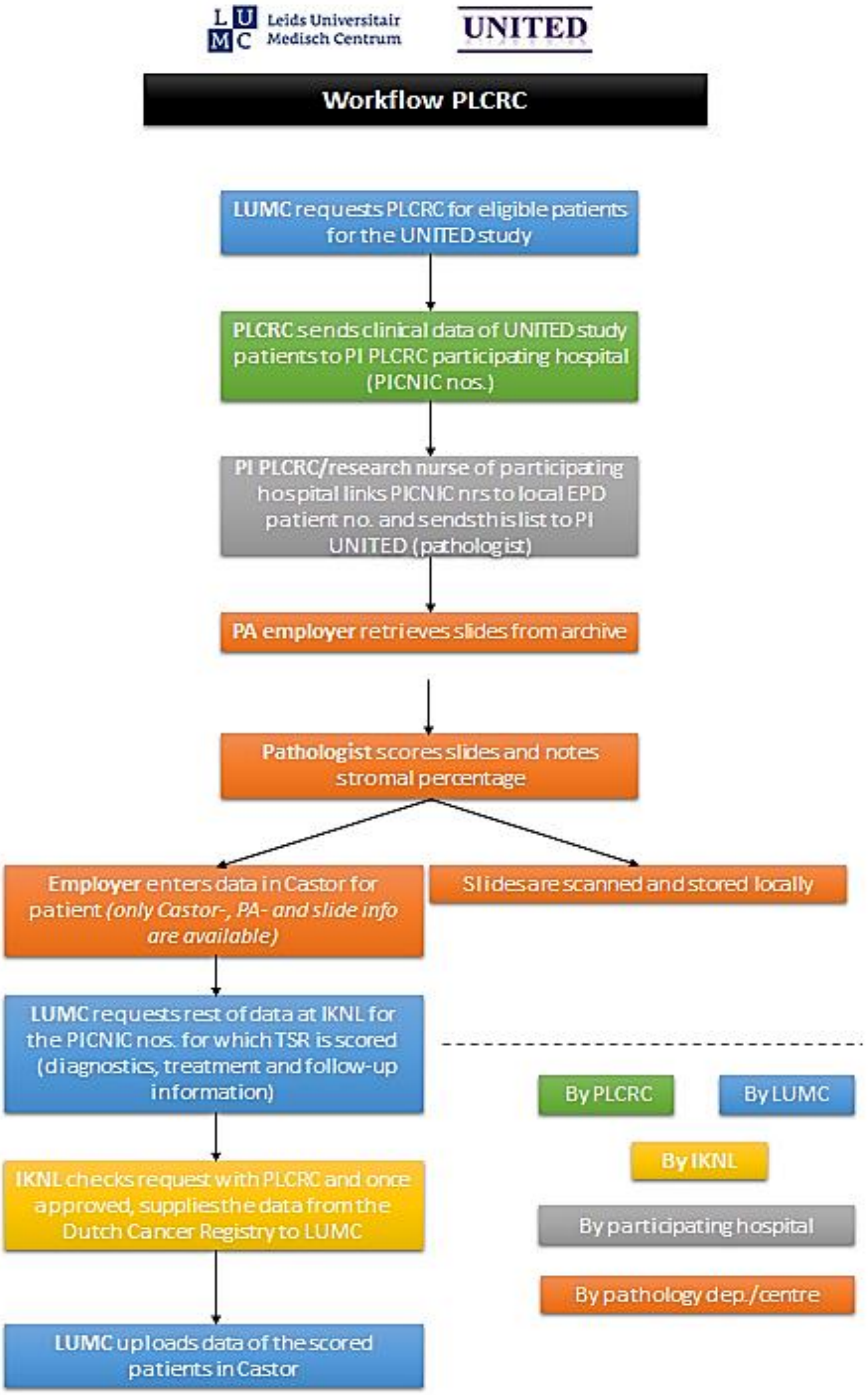

Supplementary Figure S2. Workflow patient inclusion through PLCRC collaboration.

PA, Pathology; PI, Principle investigator; PLCRC, Prospective Dutch ColoRectal Cancer cohort; LUMC, Leiden University Medical Center; IKNL, Institute for Cancer in the Netherlands.

Supplementary Table 2. Participating centres and collaborative investigators with associated inclusion rates

No. inclusions	Location (country)	Institute	Investigator (department)
230	Skopje (Macedonia)	Medical Faculty of Ss. Cyril and Methodius University	Gordana Petrushevska# (pathology)<br>Magdalena Bogdanovska (pathology)<br>Panche Zdravkoski (pathology)<br>Svetozar Antovic (surgery)<br>Darko Dzambaz (surgery)<br>Panche Karagjozov (surgery)
133	Rotterdam region (Netherlands)	PATHAN Laboratories$ *(Franciscus Gasthuis & Vlietland; Admiraal De Ruyter Ziekenhuis; IJsselland Ziekenhuis)*	Erienne M.V. de Cuba#* (pathology)<br>Frédérique Beverdam# (surgery; Franciscus Gasthuis & Vlietland)<br>Jan Jansen# (surgery; Admiraal de Ruyter Ziekenhuis)<br>Maarten Vermaas# (surgery; IJsselland Ziekenhuis)
117	Ljubljana (Slovenia)	Onkološki Inštitut	Gorana Gašljević# (pathology)
114	Vejle (Denmark)	Vejle Sygehus – Sygehus Lillebælt	Sanne Kjær-Frifeldt# (pathology)<br>Jan Lindebjerg (pathology)
111	Venlo (Netherlands)	VieCuri Medisch Centrum	Maud Strous# (pathology)<br>Jeroen F. Vogelaar (surgery)
101	Hoofddorp and Haarlem (Netherlands)	Spaarne Gasthuis$	Nicole W.J. Bulkmans#* (pathology)
83	Enschede region (Netherlands)	LabPON$ *(Medisch Spectrum Twente; Ziekenhuisgroep Twente; ZorgSaam Terneuzen)*	Joop van Baarlen# (pathology; retired)<br>Leonie Mekenkamp (medical oncology; Medisch Spectrum Twente)<br>Ronald Hoekstra (medical oncology; Ziekenhuisgroep Twente)<br>Mark Sie (medical oncology; ZorgSaam Terneuzen)
80	Barcelona (Spain)	Hospital Clinic	Miriam Cuatrecasas# (pathology)<br>Sara Simonetti (pathology)<br>María Teresa Rodrigo (pathology)<br>Iván Archilla Sanz (pathology)<br>Jose Guerrero Pineda (pathology)
75	Deventer (Netherlands)	Deventer Ziekenhuis$	Natalja E. Leeuwis-Fedorovich# (pathology)<br>Koen A. Talsma (surgery)
70	João Pessoa (Brazil)	Napoleão Laureano Hospital	Ricella M. Souza da Silva# (pathology)
57	Utrecht (Netherlands)	Universitair Medisch Centrum Utrecht$	Miangela M. Lacle# (pathology)<br>Miriam Koopman (medical oncology)
55	Delft (Netherlands)	Reinier de Graaf Gasthuis$	Jan Willem T. Dekker# (surgery)<br>Arjan van Tilburg (pathology)

53	Barcelona (Spain)	Vall d'Hebron Institute of Oncology	Paolo Nuciforo# (pathology)<br>Xenia Villalobos Alberú (pathology)<br>Stefania Landolfi (pathology)<br>Adriana Zucchiatti (pathology)
42	Alkmaar (Netherlands)	Symbiant Laboratories$ *(Noordwest Ziekenhuisgroep Alkmaar)*	Emma Witteveen# (pathology)<br>Arad Bordbar (pathology)<br>Mathijs P. Hendriks (medical oncology)
41	Amersfoort (Netherlands)	Meander Medisch Centrum$	René Arensman# (pathology)
38	Hardwick (United Kingdom)	University Hospital of North Tees	Shonali Natu# (pathology)
34	Glasgow regio (United Kingdom)	NHS Greater Glasgow and Clyde	Noori Maka# (pathology)
28	Leiden (Netherlands)	Leids Universitair Medisch Centrum	Wilma E. Mesker# (surgery)<br>Rob A.E.M. Tollenaar (surgery)<br>Meaghan Polack (surgery)<br>Marloes A. Smit (surgery)<br>Gabi W. van Pelt (surgery)<br>Hein Putter (biomedical data sciences)<br>Elma Meershoek-Kleinenbarg (clinical research center, surgery)<br>Annet G.H. Roodvoets (clinical research center, surgery)<br>Augustinus S.L.P. Crobach (pathology)<br>Hans Gelderblom (medical oncology)
28	Lisbon (Portugal)	Hospital CUF Tejo	Mário Fontes e Sousa# (medical oncology)<br>Paula Borralho Nunes (pathology)<br>João Cruz (pathology)<br>Ana Raimundo (medical oncology)<br>Nelson Silva (surgery)
24	Almada (Portugal)	Hospital Garcia de Orta	Maria J. Brito# (pathology)
19	The Hague (Netherlands)	Haaglanden Medisch Centrum	Valeska Terpstra# (pathology)
4	Kiev (Ukraine)	Bogomolets - Kyiv Oncology Center	L.M. Zakhartseva (pathology)
N/A	Brussels (Belgium)	European Society for Pathology	Raed Al Dieri (pathology)<br>Jean-François Fléjou (pathology)<br>Roger Feakins (pathology)<br>Els Dequeker (pathology)
N/A	Utrecht (Netherlands)	Netherlands Comprehensive Cancer Organisation (IKNL)$	Geraldine R. Vink (research and development)
N/A	Nijmegen (Netherlands)	Radboud University Medical Center	J. Han J.M. van Krieken (pathology)

N/A, Not applicable.

Local principal investigator.

$ Part of the PLCRC collaboration.

* Currently employed elsewhere.

Supplementary Table 3. Baseline characteristics of the total UNITED cohort

**Baseline characteristics**	**Total UNITED cohort (*N*=1,537)**
Sex	
Female	686 (45)
Male	851 (55)
Age at surgery – years	
Median age	70 (61 – 77)
≥75 years of age	509 (33)
Ineligible at registration	31 (2)
Biopsy taken	
Yes	1,353 (88)
No*	179 (12)
Rectal cancer	3 (0)
Surgery	
Surgery year	2019 (2018 – 2020)
Surgery before 2015	1 (0)
No surgery	1 (0)
Pathological TNM-stage	
Stage 0 - I	42 (3)
Stage II	756 (49)
Stage III	694 (45)
Stage IV	9 (1)
Multiple colon tumors	21 (1)
Lymph nodes - number	
Examined *(in total group)*	20 (14 – 28)
Positive *(in pTNM-stage III)***	2 (0 – 33)
Tumor-stroma ratio	
Stroma-low (≤50%)	969 (63)
Stroma-high (>50%)	433 (28)
Residual tumor	2 (0)
Missing slide	4 (0)
Follow-up	
Postoperative mortality <3 months	26 (2)
Not started follow-up	5 (0)
Withdrew consent	4 (0)
Included in final analysis	1,388 (90)

All variables are given as absolute numbers with associated percentages or medians with interquartile ranges. Sum of percentages can be less or more than 100 due to rounding.

* Reasons why biopsy was not taken, is almost always in emergency setting (obstructive ileus).
**Although there are no positive lymph nodes, using the UICC version 8, a tumor deposit (leading to stage N1c) will also lead to a pathological TNM-stage III.
TNM, Tumor-node-metastasis stage

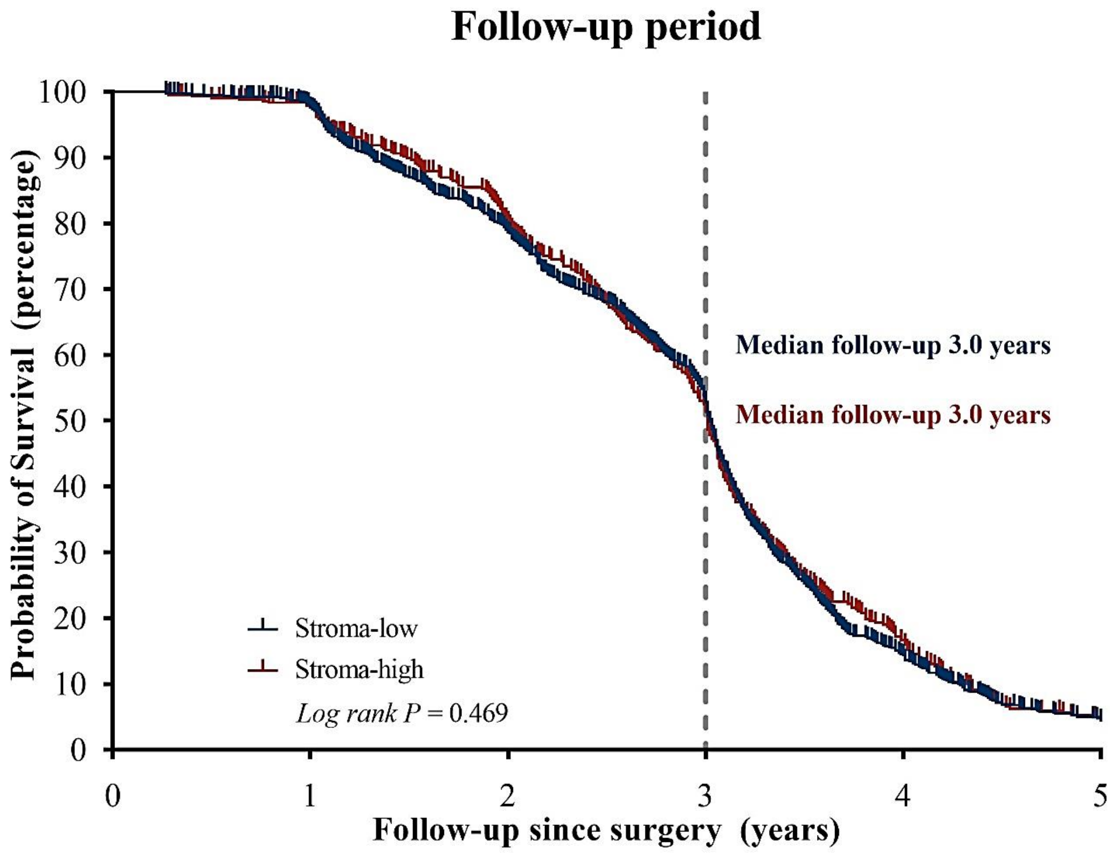

Supplementary Figure S3. Median follow-up period.

Follow-up times calculated with reverse Kaplan-Meier analysis and log rank test, showing similar follow-up curves for the stroma-high group (median follow-up 3.0 years, 95% confidence interval 2.9 – 3.1) and stroma-low (median follow-up 3.0 years, 95% confidence interval 3.0 – 3.1) (*P*=0.469).

Supplementary Table 4. General overview of patient outcomes

**Patient outcome**	**Stroma-low (*N*=960)**	**Stroma-high (*N*=428)**	***P*-value**
Follow-up time – years			0.469$
Median follow-up time	3.0 (3.0 – 3.1)	3.0 (2.9 – 3.1)	
Mutational status determined			
Not determined	864 (90)	371 (87)	
Determined, of which	96 (10)	57 (13)	0.068#
No mutations	38 (40)	16 (28)	
Mutations present, of which*	58 (60)	41 (72)	0.150#
KRAS	21 (36)	21 (51)	
BRAF	31 (53)	16 (39)	
NRAS	1 (2)	2 (5)	
PIK3A	4 (7)	2 (5)	
TP53	6 (10)	8 (20)	
Other	8 (14)	7 (17)	
Disease-free survival – years			
Disease-free survival time	5.2 (5.0 – 5.3)	4.8 (4.4 – 5.1)	**<0.001ǂ**
Stage and treatment group			**<0.001#**
Stage II - No adjuvant therapy	434 (45)	125 (29)	
Number of events	47 (11)	27 (22)	
Stage II + Adjuvant therapy	107 (11)	57 (13)	
Number of events	9 (8)	14 (25)	
Stage III + Adjuvant therapy	314 (33)	197 (46)	
Number of events	63 (21)	60 (31)	
Stage III - No adjuvant therapy	105 (11)	49 (11)	
Number of events	44 (42)	22 (45)	
Disease-free status			
No event	797 (83)	305 (71)	**<0.001#**

Event	163 (17)	123 (29)	
Type of event			**<0.001#**
No event	797 (83)	305 (71)	
Death by any cause	39 (4)	15 (4)	
Distant metastasis	105 (11)	92 (22)	
Locoregional recurrence	10 (1)	7 (2)	
Simultaneous distant metastasis and locoregional recurrence	9 (1)	9 (2)	
Location of distant metastasis			**0.007#**
Liver	37 (35)	26 (29)	
Lung	22 (21)	6 (7)	
Liver and lung	14 (13)	6 (7)	
Bone	2 (2)	1 (1)	
Brain	1 (1)	3 (3)	
Peritoneal metastases	10 (10)	12 (13)	
Abdominal lymph nodes	2 (2)	2 (2)	
Two or more locations	16 (15)	29 (32)	
Other	1 (1)	5 (6)	
Overall survival – years			
Overall survival time	5.5 (5.3 – 5.7)	5.6 (5.3 – 5.9)	0.102ǂ
Overall survival status			0.053#
Alive	858 (89)	367 (86)	
Died	102 (11)	61 (14)	
Cause of death			**0.020#**
(Metastases of) current colon cancer	58 (57)	48 (79)	
Second primary malignancy	5 (5)	4 (7)	
Other, including pre-existing comorbidity	27 (26)	6 (10)	
Missing	12 (12)	3 (5)	

All variables are given as absolute numbers with associated percentages or medians with interquartile ranges.

*Multiple mutations can occur simultaneously, hence the number of added percentages can be higher than 100 and no analysis is performed.

N/A, Not applicable.

Calculated with the Chi-square test.

$ Calculated with a reverse Kaplan-Meier analysis and log rank test.

ⱡ Calculated with Kaplan-Meier analysis and log rank test.

Supplementary Figure S4. Forest plot of the effect of risk factors on disease-free survival (univariate Cox regression analysis).

**Risk factor**	**Events/patients**	**Hazard ratio**	**95% Confidence interval (lower – upper limit)**	***P*-value**
Male sex	770 (55%)	1.04	0.82 – 1.31	0.753
Age ≥75 years	453 (33%)	1.29	1.02 – 1.63	**0.008**
No biopsy taken	165 (12%)	2.60	1.96 – 3.46	**<0.001**
Right-sided tumor	659 (47%)	0.91	0.72 – 1.14	0.404
Lymph nodes <12 examined	147 (11%)	1.54	1.11 – 2.14	**0.009**
pT4-category	273 (20%)	2.25	1.76 – 2.89	**<0.001**
pN1-category	444 (32%)	1.54	1.43 – 2.48	**<0.001**
pN2-category	221 (16%)	3.33	1.76 – 2.89	**<0.001**
Other tumor morphology*	143 (10%)	1.13	0.79 – 1.62	0.514
Poor-undifferentiated tumor grading	166 (12%)	1.25	0.90 – 1.75	0.191
Presence of ≥1 pathology risk factor**	625 (45%)	2.17	1.71 – 2.76	**<0.001**
Adjuvant chemotherapy received	675 (49%)	1.14	0.90 – 1.44	0.267
Stroma-high tumor	428 (31%)	1.78	1.41 – 2.26	**<0.001**
Microsatellite stable (MSS) tumor	587 (42%)	1.55	0.97 – 2.48	0.070

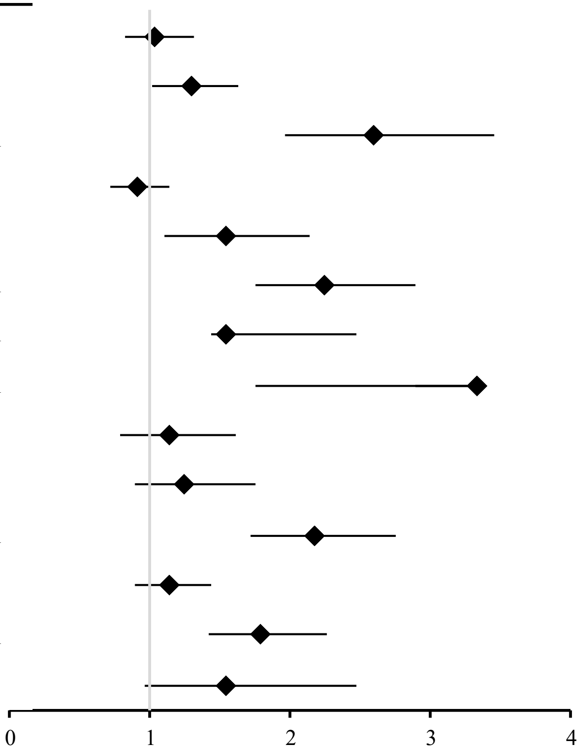

***** Other tumor morphology include signet cell carcinoma or medullary carcinoma.

** Pathology risk factors are stated below, presence of a risk factor is defined as at least one of registered risk factors.

Absence is the absence of registered risk factors, as not all risk factors are registered. Risk factors include extramural vascular invasion (EMVI), perineural invasion (PnI), etc.

Supplementary Figure S5. Forest plot of the effect of risk factors on disease-free survival using significant variables from the univariate analysis (multivariate Cox regression analysis).

Risk factor	Events/patients	Hazard ratio	95% Confidence interval (lower – upper limit)	*P*-value
Age ≥75 years	453 (33%)	1.54	1.21 – 1.96	**<0.001**
No biopsy taken	165 (12%)	2.33	1.72 – 3.16	**<0.001**
Lymph nodes <12 examined	147 (11%)	1.38	0.98 – 1.95	0.067
pT4-category	273 (20%)	1.59	1.22 – 2.07	**<0.001**
pN1-category	444 (32%)	1.71	1.28 – 2.30	**<0.001**
pN2-category	221 (16%)	2.66	1.91 – 3.70	**<0.001**
Presence of ≥1 pathology risk factor*	625 (45%)	1.38	1.06 – 1.81	**0.018**
Stroma-high tumor	428 (31%)	1.49	1.17 – 1.90	**0.001**

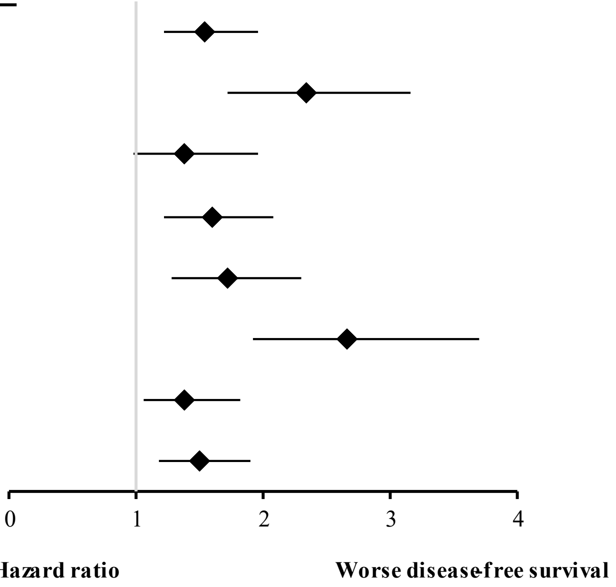

* Pathology risk factors are stated below, presence of a risk factor is defined as at least one of registered risk factors.

Absence is the absence of registered risk factors, as not all risk factors are registered. Risk factors include extramural vascular invasion (EMVI), perineural invasion (PnI), etc.

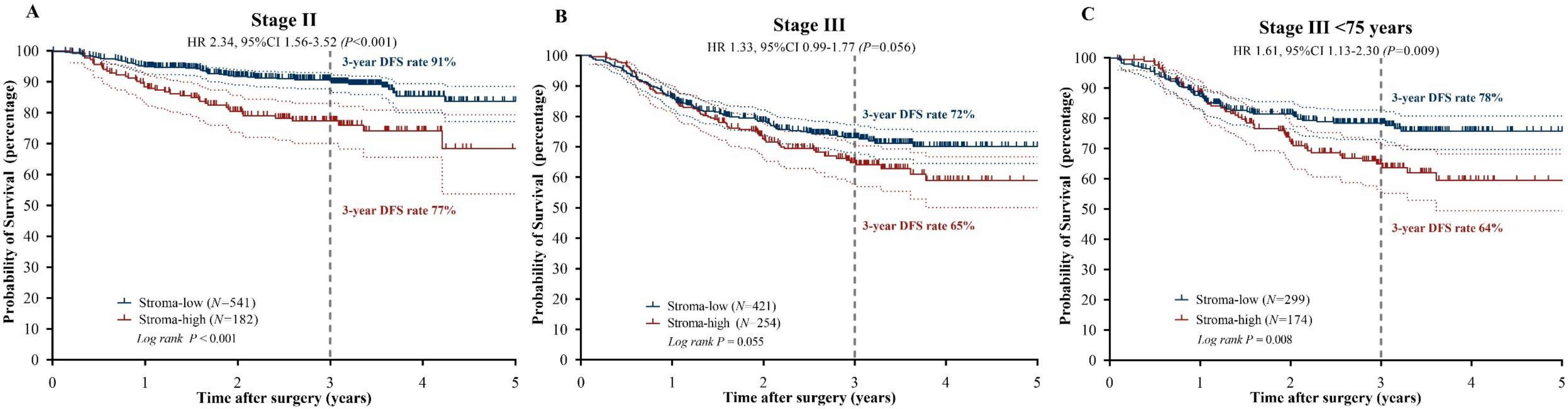

Supplementary Figure S6. TSR on Disease-free survival per TNM-stage.

Kaplan-Meier curve and log rank analysis of TSR category and plotted 95% confidence intervals. A) Stage II with 3-year survival rates of 77% vs. 91%, respectively (*P*<0.001); B) Stage III, with 3-year survival rates of 65% vs. 72%, respectively (*P*=0.055). TNM-stage III is only nearly significant due to bias through high number of patients with comorbidities or high age and not treated with standard adjuvant chemotherapy. C) After stratifying for age <75 years, stage III stroma-high colon cancer also leads to significantly worse disease-free survival, with 3-year rates of 64% vs. 78% (*P*=0.008). TNM, Tumor-node-metastasis stage; TSR, Tumor-stroma ratio.

Supplementary Table 5. Detailed overview of the adjuvant chemotherapy regimens and durations

**Variables**	**Total eligible (*N*=1,388)**	**Stroma-low (*N*=960)**	**Stroma-high (*N*=428)**	***P*-value**
Adjuvant chemotherapy – not started				
Total not started adjuvant treatment	713 (51)	539 (56)	174 (41)	**<0.001#**
Stage II	559 (78)	434 (80)	125 (72)	**<0.001#**
Stage III	154 (22)	105 (20)	49 (28)	**<0.001#**
Reasons not started adjuvant treatment*				N/A
Not indicated	509 (71)	401 (76)	108 (62)	
Comorbidity, age	100 (14)	70 (14)	30 (17)	
Other, including patients wish	88 (16)	56 (10)	32 (18)	
Missing	20 (3)	15 (3)	5 (3)	
Adjuvant chemotherapy – started				
Total started treatment	675 (49)	421 (44)	254 (59)	**<0.001#**
Stage II	164 (24)	107 (26)	57 (22)	**<0.001#**
Stage III	511 (76)	314 (74)	197 (78)	**<0.001#**
Regimen started adjuvant chemotherapy**				N/A
CAPOX/XELOX***	394 (58)	238 (57)	156 (61)	
Capecitabine monotherapy	173 (26)	112 (27)	61 (24)	
FOLFOX	33 (5)	26 (6)	7 (3)	
Other, including 5FU monotherapy	32 (5)	18 (4)	14 (6)	
Missing	43 (6)	27 (6)	16 (6)	
Duration adjuvant chemotherapy – months				0.118$
Median duration	3 (2 - 5)	3 (2 - 5)	2 (2 - 5)	
Missing	52 (8)	32 (8)	20 (8)	

All variables are given as absolute numbers with associated percentages or medians with interquartile ranges.

*Multiple reasons can occur, hence the number of added percentages can be higher than 100. The first given reason is shown here.

**Switch in regimens can occur, hence the number of added percentages can be higher than 100. The initially started regimen is mostly shown here.

***CAPOX/XELOX regimens here are both the 3 and 6 cycles.

5FU, 5-Fuorouracil intravenous chemotherapy; CAPOX/XELOX, Oral capecitabine with intravenous oxaliplatin chemotherapy; FOLFOX, Intravenous 5-fluorouracil and oxaliplatin; TNM, Tumor-node-metastasis stage.

Calculated with the Chi-square test.

$ Calculated with an Independent Student's T-test.

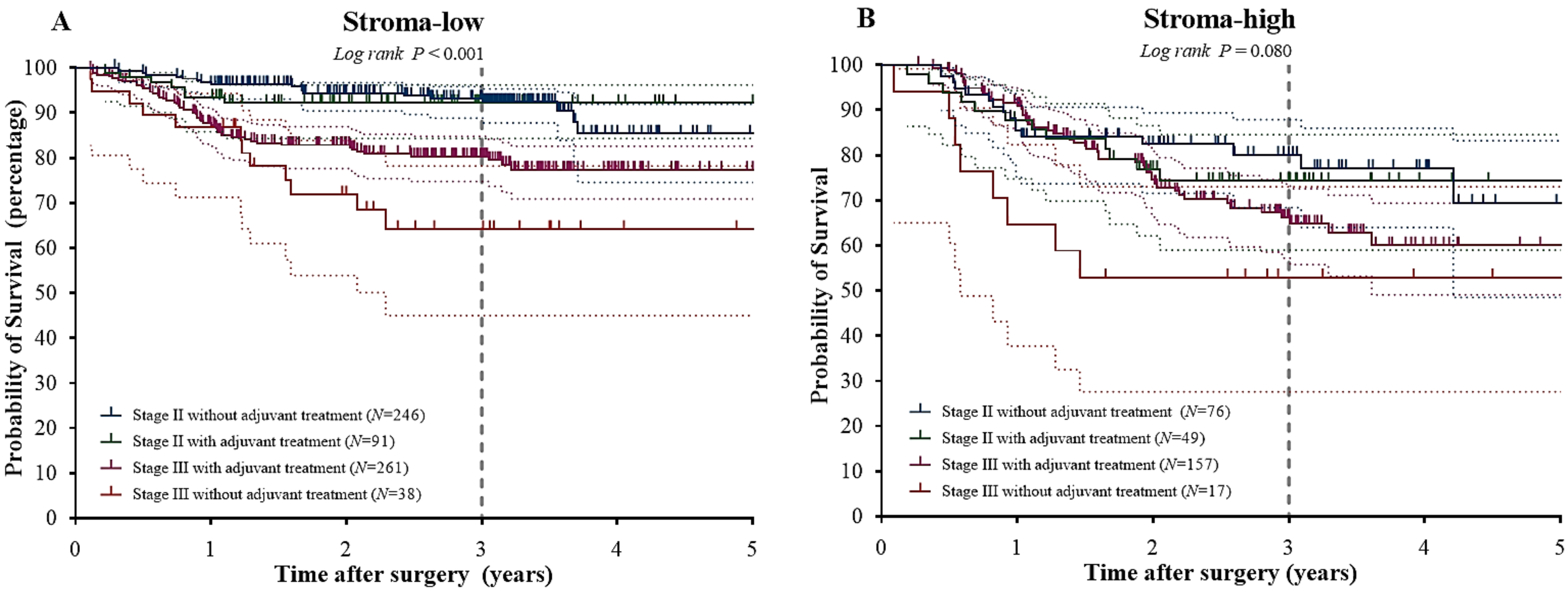

Supplementary Figure S7. Disease-free survival per TSR category and benefit of adjuvant treatment per TNM-stage.

Kaplan-Meier curve and log rank analysis of TSR category and plotted 95% confidence intervals. A) Stroma-low patient groups showing an significant influence of adjuvant treatment, with 3-year survival rates of 93% vs. 92% vs. 80% vs. 64%, respectively (*P*<0.001); B) Stroma-high patient groups showing worse outcomes than stroma-low groups in A but also within groups no significant difference despite adjuvant treatment, with 3-year survival rates of 80% vs. 73% vs. 66% vs. 52% (*P*=0.080). In TNM-stage III patients not receiving adjuvant treatment, bias occurs e.g. due to small numbers. TNM, Tumor-node-metastasis stage; TSR, Tumor-stroma ratio.

Supplementary Table 6. Overview of ASCO criteria and TSR categories

Variables	Total no. (%)	No. events (%)	*P*-value
Stage II - No adjuvant chemotherapy			**<0.001#**
Patients <75 years of age*	322 (100)	35 (11)	
Tumor-stroma ratio			**0.001#**
Stroma-low	246 (76)	19 (8)	
Stroma-high	76 (24)	16 (21)	
ASCO-criteria**			0.383#
ASCO low-risk	199 (62)	24 (12)	
ASCO high-risk	123 (38)	11 (9)	

All variables are given as absolute numbers with associated percentages or medians with interquartile ranges.

*Compared to the category as defined in Supplementary Table 4.

**ASCO-criteria include a pT4 tumor, sampling of <12 lymph nodes or emergency setting of surgery, presence of pathological risk factors like lymphovascular or perineural invasion, and poor tumor differentiation as risk factors. If one is present, categorization as ASCO high-risk followed.

ASCO, American Society for Clinical Oncology; TSR, Tumor-stroma ratio.

Calculated with the Chi-square test.

Supplementary Table 7. Data sharing statement

**Data sharing statement**	
Will individual participant data be made available (including data dictionaries)?	Yes, after approval by the Steering Committee
What data in particular will be shared?	All of the individual participant data collected during the study after de-identification
Additional information regarding the data:	Data on surgery, pathology, chemotherapy, follow-up and survival
Which other documents will be made available?	Study protocol, publications
When will the data be available (start and end dates)?	Start data of availability is after the end of 2023, after finishing manuscripts written by the researchers and others from the Steering Committee. There is no end date
To whom will the data be available?	Qualifying researchers who submit a proposal with a valuable research question, as agreed upon by the Steering Committee
For what types of analyses?	To achieve aims in the approved proposal
By what mechanism will data be made available?	Research proposals should be directed to associate professor Wilma Mesker, principal investigator of the UNITED study, at w.e.mesker@lumc.nl. Data requestors will need to sign a Data Sharing Agreement
